# Connexin and pannexin hemichannels in brain glial cells: properties, pharmacology, and roles

**DOI:** 10.3389/fphar.2013.00088

**Published:** 2013-07-17

**Authors:** Christian Giaume, Luc Leybaert, Christian C. Naus, Juan C. Sáez

**Affiliations:** ^1^Collège de France, Center for Interdisciplinary Research in Biology/Centre National de la Recherche Scientifique, Unité Mixte de Recherche 7241/Institut National de la Santé et de la Recherche Médicale U1050Paris, France; ^2^University Pierre et Marie CurieParis, France; ^3^MEMOLIFE Laboratory of Excellence and Paris Science Lettre Research UniversityParis, France; ^4^Physiology Group, Department of Basic Medical Sciences, Faculty of Medicine and Health Sciences, Ghent UniversityGhent, Belgium; ^5^Department of Cellular and Physiological Sciences, Life Sciences Institute, Faculty of Medicine, University of British ColumbiaVancouver, BC, Canada; ^6^Departamento de Fisiología, Pontificia Universidad Católica de ChileSantiago, Chile; ^7^Instituto Milenio, Centro Interdisciplinario de Neurociencias de ValparaísoValparaíso, Chile

**Keywords:** astrocytes, oligodendrocytes, microglia, gap junctions, neuroglial interactions

## Abstract

Functional interaction between neurons and glia is an exciting field that has expanded tremendously during the past decade. Such partnership has multiple impacts on neuronal activity and survival. Indeed, numerous findings indicate that glial cells interact tightly with neurons in physiological as well as pathological situations. One typical feature of glial cells is their high expression level of gap junction protein subunits, named connexins (Cxs), thus the membrane channels they form may contribute to neuroglial interaction that impacts neuronal activity and survival. While the participation of gap junction channels in neuroglial interactions has been regularly reviewed in the past, the other channel function of Cxs, i.e., hemichannels located at the cell surface, has only recently received attention. Gap junction channels provide the basis for a unique direct cell-to-cell communication, whereas Cx hemichannels allow the exchange of ions and signaling molecules between the cytoplasm and the extracellular medium, thus supporting autocrine and paracrine communication through a process referred to as “gliotransmission,” as well as uptake and release of metabolites. More recently, another family of proteins, termed pannexins (Panxs), has been identified. These proteins share similar membrane topology but no sequence homology with Cxs. They form multimeric membrane channels with pharmacology somewhat overlapping with that of Cx hemichannels. Such duality has led to several controversies in the literature concerning the identification of the molecular channel constituents (Cxs versus Panxs) in glia. In the present review, we update and discuss the knowledge of Cx hemichannels and Panx channels in glia, their properties and pharmacology, as well as the understanding of their contribution to neuroglial interactions in brain health and disease.

## INTRODUCTION

For a long time, it has been taken as dogma that connexin (Cx) proteins can only function as gap junction channels. Indeed, before the aggregation of Cxs at the junctional plaque and subsequent formation of gap junctions, hexameric rings of Cxs, termed connexons, were initially assumed to remain closed. An obvious reason for this occlusion was that, as gap junction channels are “poorly” selective for ions and permeable to low molecular weight molecules (<1 to 1.2 KDa), if once at the membrane connexons could open, the cell would lose its integrity or at least would have to spend substantial energy to maintain this energetically unfavorable condition. Such statements began to be challenged when evidence for “functional hemichannels,” a term proposed to substitute for plasma membrane connexons, were reported in the early 1990s. In these pioneering studies, Cx hemichannels were opened either by large depolarization in *Xenopus* oocytes (Paul et al., 1991) or by lowering the extracellular calcium ion (Ca^2+^) concentration in horizontal cells (DeVries and Schwartz, 1992). Later on, the occurrence of functional hemichannels (i.e., hemichannels that can be turned into the open state) composed of Cx43 was demonstrated in primary cultures of astrocytes in the absence of external Ca^2+^ (Hofer and Dermietzel, 1998). This observation was followed by the demonstration that metabolic inhibition performed in the presence of normal external Ca^2+^ concentrations (1–2 mM) induced cell permeabilization, due to Cx43 hemichannel opening, before loss of membrane integrity (Contreras et al., 2002). More recently, hemichannel opening in astrocytes was triggered either by treatment with pro-inflammatory cytokines or selective lipopolysaccharide (LPS) stimulation of microglia co-cultured with astrocytes, again in the presence of external Ca^2+^ (Retamal et al., 2007a). The opening of Cx43 hemichannels in astrocytes was also demonstrated to occur in experiments designed to decipher the mechanism of intercellular Ca^2+^ wave propagation in astrocytes to which both gap junction channels and hemichannels contribute (Scemes and Giaume, 2006; Leybaert and Sanderson, 2012). In this case, the release of ATP through Cx43 hemichannels turned out to play a key role in Ca^2+^ wave propagation mediated by an extracellular paracrine signaling component (Cotrina et al., 1998; Arcuino et al., 2002). All these initial experiments on Cx hemichannels utilized astrocytes as a cell model giving to these cells new roles in neuroglial interaction (Bennett et al., 2003).

At the turn of the century another membrane channel family, the pannexins (Panxs), was identified (Panchin et al., 2000) and demonstrated to be orthologs of innexins, the gap junction proteins expressed in invertebrates (Baranova et al., 2004). So far, there are no reports of gap junctional communication supported by native Panxs, although over expression of Panx1 enhances gap junctional coupling in glioma cells (Lai et al., 2007). They form membrane channels contributing to membrane permeabilization, similar to Cx hemichannels, and they are expressed in glial cells (Orellana et al., 2009; Scemes, 2012). The aim of the present review is to summarize what is actually known about Cx hemichannels and Panx channels in glia (mainly astrocytes and microglia) and to discuss their contribution to neuroglial interactions taken in the normal and pathological context. An overview of methods and approaches to investigate Cx hemichannels and Panx channels has recently been published (Giaume et al., 2012); we refer the reader to this paper for a more detailed discussion of methodological aspects.

## CONNEXIN AND PANNEXIN EXPRESSION IN GLIAL CELLS

**FIGURE 1:**
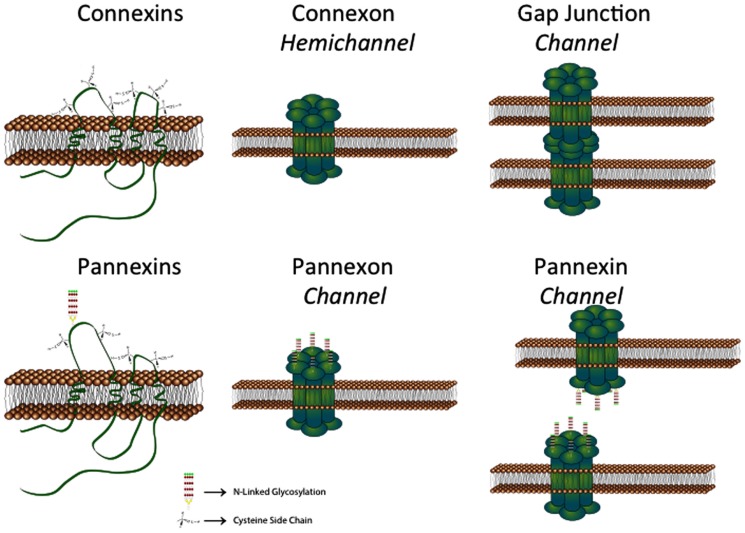
**Schematic showing that connexins can from both hemichannels and gap junction channels. In contrast, pannexins normally form channels equivalent to hemichannels. See text for details (provided by Stephen Bond, Ph.D., UBC)**.

Gap junctions provide a unique direct conduit between cells, influencing crucial cellular processes through activities including electrical conductance and metabolic cooperation, mediated by the passage of ions, amino acids, glucose, glutathione, ATP, and small signaling molecules (reviewed in Sohl and Willecke, 2004), as well as microRNAs (Katakowski et al., 2010). When one considers Cx expression in the brain (Theis et al., 2005), it is evident that they are most abundant in glia, namely the astrocytes, oligodendrocytes, and microglia (Rouach et al., 2002). With the genomic characterization of Cx (Willecke et al., 2002) and Panx (Baranova et al., 2004) gene families, it has been determined that there are at least 10 Cxs and 2 Panxs expressed in the brain (see below). There is also a temporal (i.e., developmental) and spatial (i.e., cell type and brain region) pattern regarding this expression and localization. Furthermore, these channel proteins can have different roles in different cell types, either as the canonical gap junction intercellular channel or as a hemichannel (Goodenough and Paul, 2003; **Figure 1**). Cx hemichannels and Panx1 channels mediate the release of ATP that activates P2 receptors, promoting rises in intracellular Ca^2+^ concentrations; gap junction channels mediate the intercellular transfer of second messenger propagated calcium signal (Suadicani et al., 2004; Anselmi et al., 2008; Leybaert and Sanderson, 2012). Under pathological conditions, Panx1 channels have been proposed to mediate activation of the inflammasome both in neurons and astrocytes (Iglesias et al., 2009; Silverman et al., 2009), whereas Cx43 hemichannels are involved in acceleration of astroglia and neuronal cell death triggered by hypoxia-reoxygenation in high glucose (Orellana et al., 2010) and β-amyloid peptide, respectively (Orellana et al., 2011b,c). A further level of complexity can be envisioned in the context of Cxs, where the heteromeric assembly of different Cx proteins into a single channel can result in distinct differences in biophysical properties (Bevans et al., 1998; Harris, 2007). This feature is also true for Panxs since Panx1 can form both homomeric and heteromeric channels with Panx2 (Bruzzone et al., 2005). Thus for specific cellular functions, the complement of Cxs and Panxs expressed is important in the physiological or pathological context being considered. While other “non-channel” functions are emerging for Cxs (Vinken et al., 2012) and Panxs (MacVicar and Thompson, 2010), this review will be limited to channel-related functions and properties.

While this review will focus on gap junction channels and hemichannels in brain glial cells, it is important to note that gap junction proteins have also been identified in brain neurons (Andrew et al., 1981; Söhl et al., 2005). These include Cx26, Cx30.2, Cx36, Cx45, and Cx57 (Rouach et al., 2002). Neurons also express Panx1 *in vivo* (Bruzzone et al., 2003), and recently it has been reported that neural progenitors in the adult rodent hippocampus express Panx2 (Swayne et al., 2010). It is thus possible, and has been reported in some situations, that neurons form gap junctions with astrocytes since some of the same Cxs are expressed in both cell types (Fróes et al., 1999; Alvarez-Maubecin et al., 2000). Astrocytes express multiple Cxs, including Cx43, Cx26, Cx30, Cx40, Cx45, and Cx46 (Giaume et al., 2012), as well as Panx1 (Iglesias et al., 2009) and Panx2 (Zappala et al., 2007). Non-treated microglia express Cx43 (Eugenín et al., 2001; Garg et al., 2005), Cx36 (Dobrenis et al., 2005), and Cx32 (Maezawa and Jin, 2010), as well as Panx1 (Orellana et al., 2011b). Finally, oligodendrocytes express Cx32, Cx47, and Cx29 *in vivo* (Altevogt et al., 2002; Wasseff and Scherer, 2011). The complexity of Cxs and Panxs functioning as gap junction channels/hemichannels and channels, respectively, in these various cell types can be viewed as providing a background level of activity to support and modulate the complex neural activity mediated *via* gliotransmission.

The expression profiles noted have been associated with mature neurons and glial cell types. However, these various cell types arise developmentally from neuronal and glial precursors. Several studies have demonstrated changes in expression of Cxs and Panxs during brain development (reviewed in Vogt et al., 2005; Belousov and Fontes, 2013). Given that several reports have described disturbed neural development when expression of some Cxs is modified through the use of gene knockdown or knockout strategies, gap junction channels and hemichannels are likely to play significant roles in processes including cell proliferation, migration, and differentiation (Fushiki et al., 2003; Elias et al., 2007; Wiencken-Barger et al., 2007; Cina et al., 2009; Liu et al., 2012).

Initial studies on Cx functions in neural cells in culture and in brain slices focused on their expected role in forming gap junction channels, confirming their function *via* electrophysiological analysis or methods using low molecular weight dye tracers, which can pass from cell to cell through these intercellular channels (Giaume et al., 1991). Therefore, many of the phenotypic changes observed when enhancing or decreasing Cx expression were attributed to changes in gap junctional communication. With the recognition that Cxs can also form hemichannels, the consequences of Cx expression began to be considered in a new way (Contreras et al., 2004), and appropriate methods developed to assess their presence and activity (Giaume and Theis, 2010). Cx hemichannels and Panx channels are more typical of most membrane channels, like ion channels, in their direct communication between intracellular and extracellular space. However, they are unique in their pore size and conductance properties (Thompson and Macvicar, 2008).

The formation of gap junction channels and hemichannels by Cxs can help explain some contradictory effects of altering Cx expression. For example, enhanced Cx43 expression in brain glial cells, in culture and *in vivo*, has been shown to be both neuroprotective and neurodestructive in ischemic and excitotoxic situations (reviewed in Orellana et al., 2009; Kozoric and Naus, 2013). Recent studies have indicated that gap junction channels formed by Cx43 can be neuroprotective, while Cx43 hemichannels may be more detrimental to neuronal survival. The following sections of this review provide further insight into the distinct properties of hemichannels and how these impact the progression of neurological disorders.

## Cx HEMICHANNEL AND Panx CHANNEL PROPERTIES IN GLIA

### BIOPHYSICS

Today, most Cxs expressed in rodent glial cells, including Cx26, Cx30, Cx32, Cx43, and Cx45, have been shown to form functional hemichannels in endogenous and/or exogenous expression systems (Bennett et al., 2003). Nevertheless, it remains unknown if this is also the case for Cx29 and Cx47 found in oligodendrocytes. In exogenous expression systems, Cx30, Cx32, Cx43, and Cx45 hemichannels show a very low open probability at resting membrane potential and normal concentration of the extracellular divalent cations, Ca^2+^ and Mg^2+^. For Cx26 hemichannels, these properties are species-specific; human and sheep but not rat Cx26 forms hemichannels that at resting membrane potential and normal concentration of extracellular divalent cations show opening activity but the rat protein is characterized by a single evolutionary amino acid change (D159N) that limits this feature (González et al., 2006).

Opening of Cx hemichannels studied so far can be promoted by positive transmembrane voltages and reduced concentrations of extracellular divalent cations (Bennett et al., 2003). These conditions have been most frequently used to gain knowledge on biophysical features of hemichannels, including permeability properties (see below), identification of voltage sensitivity, unitary conductance, kinetic properties, and polarity of closure. However, other gating mechanisms can also promote opening of hemichannels, as described below (see Regulation). In general, hemichannels show a time- and voltage-dependent macroscopic current, activating with depolarization and inactivating with hyperpolarization. However, this feature is not equal in hemichannels formed by different Cxs. For example, the voltage dependency is strong for mouse Cx30 hemichannels (Valiunas and Weingart, 2000) but detectable only at very high positive potentials for rat Cx43 hemichannels (Contreras et al., 2003). For Cx32 hemichannels, the conductance is determined by charges dispersed over the pore pathway (Oh et al., 2008). Hemichannels formed by human Cx26 or rat Cx43 show bipolar voltage behavior (Contreras et al., 2003; González et al., 2006); however, this appears to be less well established for Cx43, for which unitary current events were found to monotonically rise in the voltage range of +30 to +90 mV (Wang et al., 2012). Hemichannels present two distinct voltage-gating mechanisms, which are called loop- (slow) and V(j)- (fast) gating, respectively (Contreras et al., 2003; Oh et al., 2008). For Cx32 hemichannels the loop-gate is mediated by a conformational change that reduces the pore diameter in a region located close to the first transmembrane domain and first extracellular loop (Bargiello et al., 2012). It was recently also proposed that the intracellular pore entrance narrows from 15 to 10 Å with loop-gated but not with *V*(j)-gated channel closure. Moreover, it was shown that the extracellular entrance does not undergo evident large conformational changes with either voltage-gating mechanisms (Kwon et al., 2013). The unitary conductance is characteristic for hemichannels with specific Cx composition; the fully open state for Cx26 hemichannels is characterized by ~320 pS unitary conductance (González et al., 2006), for Cx32 hemichannels this is ~90 pS, for Cx43 hemichannels ~220 pS (Contreras et al., 2003; Retamal et al., 2007a; Wang et al., 2012) and for Cx45 hemichannels ~57 pS (Valiunas, 2002). They also present conductance substates as a result of an inactivation mechanism that hinders complete activation of hemichannels under prolonged depolarization (Valiunas, 2002; Contreras et al., 2003; Gómez-Hernández et al., 2003). The most general features mentioned above have been useful to demonstrate and understand the involvement of hemichannels in different physiologic functions and also to identify hemichannels with specific Cx composition in glial cells (Orellana et al., 2011c). However, much more remains to be investigated since Cxs are post-translationally modified and thus, their biophysical features might be affected according to the metabolic state of the cell.

The biophysical properties of Panx channels are much less documented than those of Cx hemichannels, possibly as a result of their more recent discovery. Panx1 channels have been so far demonstrated to be present in microglia, astrocytes, and oligodendrocytes (Iglesias et al., 2009; Domercq et al., 2010; Orellana et al., 2011a), whereas Panx2 is expressed in post-ischemic astrocytes (Zappala et al., 2007). The open state of Panx1 channels is promoted by positive membrane voltages and elevation of intracellular free Ca^2+^ concentration. Voltage clamp experiments have indicated widely divergent unitary conductances for Panx1 channels, varying from 68 to 550 pS (Locovei et al., 2006; Kienitz et al., 2011; Ma et al., 2012). This controversy in the single channel conductance of Panx1 channels has not been resolved yet and might be explained in part by differences in recording conditions or solutions, cell type, phenotypic differences, and post-translational modifications. It could be partially explained by simultaneous opening of channels and thus, high conductance values might represent multiples of a single channel with a lower conductance value. An issue to keep in mind is that biophysical properties of exogenously expressed Panx channels might be different from those expressed simultaneously with all membrane and intracellular proteins (e.g., P2X receptors) that might interact with them in endogenous expression systems.

### PERMEABILITY

In addition to selective ion channels or transporters, other channels can alter the permeability of the cell membrane under physiological and pathological conditions (Schalper et al., 2009). The origin of these permeability changes could be Cx- and Panx-based channels located at the cell surface. However, other membrane channels might also be involved in drastic changes in membrane permeability [e.g., P2X_7_ receptors, transient receptor potential (TRP) channels and calcium homeostasis modulator 1 (CALHM1) ion channel] and their possible involvement should be proved or disproved when testing the permeability of Cx hemichannels or Panx channels in a particular cell type.

Net changes in membrane permeability due to hemichannels have been most frequently studied through the use of inhibitors or molecular biology approaches that induce or abolish the expression of Cxs or Panxs. With these experimental approaches it has been proposed that hemichannels (Panx1 and/or Cx26, Cx32, and Cx43) allow the release of precursor or signaling molecules [e.g., including NAD^+^, ATP, adenosine, inositol trisphosphate (IP_3_), and glutamate; Bevans et al., 1998; Stout et al., 2002; Ye et al., 2003; Anderson et al., 2004; Bao et al., 2004a; Locovei et al., 2006; Gossman and Zhao, 2008; Kang et al., 2008; Song et al., 2011], uptake of second messengers [e.g., cyclic ADP-ribose (cADPR), nicotinic acid adenine dinucleotide phosphate (NAADP), nitric oxide (NO), and Ca^2+^; Heidemann et al., 2005; Sánchez et al., 2009, 2010; Schalper et al., 2010; Song et al., 2011; De Bock et al., 2012; Figueroa et al., 2013) and uptake of various metabolites (e.g., glucose, glutathione, and ascorbate; Bruzzone et al., 2001; Ahmad and Evans, 2002; Rana and Dringen, 2007; Retamal et al., 2007a; see **Table 1** for Cx43 hemichannels and Panx1 channels). In addition, it has been demonstrated that Cx hemichannels are permeable to synthetic molecules [e.g., Lucifer yellow, ethidium, calcein, propidium, 5(6)-carboxyfluorescein, and 2-(4-amidinophenyl)-1H-indole-6-carboxamide (DAPI); for review, see Sáez et al., 2010]. The fact that Cx26 and Cx32 hemichannels reconstituted in lipid membranes are permeable to Ca^2+^ and cyclic nucleotides, respectively (Harris, 2007; Schalper et al., 2010; Fiori et al., 2012), indicates that no additional binding partners or associated molecules are required for hemichannels to function as conduits for small molecules or ions across the cell membrane. Since the membrane permeability change due to transient hemichannel opening may allow release of enough molecules to reach effective concentrations in a confined extracellular space, astroglial hemichannels have been proposed as membrane pathways for paracrine/autocrine cell signaling with physiological and pathological implications for glial and neuronal cells (Orellana et al., 2011c; Stehberg et al., 2012).

**Table 1:** Overview of substances passing through glial Cx hemichannels and Panx channels and compounds that block these membrane pathways.

Models	Techniques	Permeant metabolic or signaling molecules	Hemichannel blocking condition used	Reference
Astrocyte cultures	Dye uptake	2-NBDG (uptake)	**Cx43**, lanthanum (La^3+^), Gap27	Retamal et al. (2007a)
Astrocyte cultures	Colorimetric Tietze method	Glutathione (release)	Divalents, alcohols, flufenamic acid, carbenoxolone	Rana and Dringen (2007)
Astrocyte cultures	HPLC analysis	Glutamate, aspartate (release)	Divalents, alcohols, flufenamic acid, carbenoxolone	Ye et al. (2003)
Acute hippocampal slices, C6 transfected cells	Bioluminescence ATP imaging	ATP (release)	**Cx43**, non-transfected C6 cells	Cotrina et al. (1998); Kang et al. (2008)
Astrocyte cultures	Luciferin/luciferase assay	ATP (release)	**Panx1**, Panx1 knock out mouse	Suadicani et al. (2012)
Microglia cultures treated with amyloidβ	Luciferin/luciferase bioluminescence and enzyme-linked fluorimetric assays	ATP, glutamate (release)	**Panx1 and Cx43**, {}^10^Panx1, probenecid	Orellana et al. (2011b)
Microglia cultures treated with TNF-α	Colorimetric assay	Glutamate (release)	**Cx32**, Gap24, carbenoxolone	Takeuchi et al. (2006)

*This table summarizes most if not all data available on signaling molecules passing through Cx or Panx channels (in bold, column 4) and conditions or blockers of the Cx hemichannels or Panx channels used in each particular study. Citations refer to the initial demonstration of the channel permeation property. The model, method of detection of the permeant compound, and reference where these findings were described are also included. 2-NBDG [2-(*N*-(7-nitrobenz-2-oxa-1,3-diazol-4-yl)amino)-deoxyglucose] is a fluorescent glucose analog.*

Membrane permeability changes *via* Cx hemichannels are in part explained by variations in the number of hemichannels available at the cell surface. With regard to this, an increase in the number of surface Cx43 or Cx45 hemichannels explains the increase in membrane permeability induced by fibroblast growth factor-1 (FGF-1) in cells that express these proteins (Schalper et al., 2008b). However, in other conditions such as exposure to extracellular solution without divalent cations (Schalper et al., 2008a) or tanycytes treated with extracellular glucose (Orellana et al., 2012a), the increase in membrane permeability is explained by opening of hemichannels *via* a gating mechanism. In both cases there is a net increase in membrane permeability mediated by hemichannels, possibly without changes in intrinsic permeability properties of hemichannels themselves. Nonetheless, changes in membrane permeability could also result from alterations in permeability features of hemichannels. For instance, protein kinase C (PKC)-mediated phosphorylation of the six subunits of Cx43 hemichannels reconstituted in lipid membranes reduces the pore permeability allowing permeation of the small hydrophilic solute ethylene glycol (Mr 62) but not sucrose (Mr 342; Bao et al., 2007). Changes in other hemichannel permeability properties, such as affinity or charge selectivity induced by covalent modification including phosphorylation by other protein kinases or nitrosylation, cannot be ruled out yet.

The permeability properties depend on the size, shape, and net charge of each permeant ion or molecule while the concentration gradient across the cell membrane acts as the driving force. Since the study of permeability characteristics of a membrane transporter should be done with a rapid time scale (within a few seconds to a minute) it is important to have a library of permeant molecules that allow real time determination of their disappearance or appearance. Transport via Cx43 hemichannels has been shown to be cooperative and saturable with parameters (e.g., *V*_max_, *K*_m_, and Hill coefficient) depending on the permeant cationic species (Orellana et al., 2011a). These findings imply that opening of hemichannels does not necessarily lead to loss of important intracellular molecules because of competition effects with other, less important molecules or ions and this interpretation might be valid to transport in both directions across the cell membrane. Of note, the permeability properties of hemichannels to anionic molecules have not been reported yet. To the best of our knowledge, the permeability properties of Panx1 channels remain equally unknown. It is also not known whether channels formed by different Cxs or Panxs show different permeability properties such as size exclusion and charge selectivity. In general, quantitative kinetic properties of permeation of Cx hemichannels and Panx channels remain largely unexplored. **Table 1** summarizes current evidence on the permeability of Cx43, Cx32 hemichannels, and Panx1 channels in brain glial cells.

### REGULATION

Hemichannels are regulated by diverse conditions of the extracellular and intracellular microenvironments. They include Ca^2+^ concentration outside and inside the cell, monovalent cation concentration, pH, mechanical stress, extracellular ligands, protein kinases, protein phosphatases, as well as oxidant and reducing agents. Here, the most recent studies not included in previous reviews (Sáez et al., 2010; Wang et al., 2013), will be presented.

#### Hemichannel opening and modulation by Ca^2+^

Unapposed Cx hemichannels of cells in culture are preferentially closed but can be opened by several trigger conditions (reviewed in Sáez et al., 2005, 2010). These include, transmembrane voltage with activation threshold of -30 mV for Cx30 and +50 mV for Cx43 in the presence of normal extracellular Ca^2+^ (Valiunas and Weingart, 2000; Wang et al., 2012), mechanical forces/strain (Bao et al., 2004b; Luckprom et al., 2011), a decrease of extracellular Ca^2+^ (Li et al., 1996; Stout et al., 2002; Ye et al., 2003; Ramachandran et al., 2007; Torres et al., 2012), an increase of intracellular free Ca^2+^ (De Vuyst et al., 2006, 2009; Wang et al., 2012), alterations in phosphorylation status (Bao et al., 2004c) and redox status (Retamal et al., 2007b), and ischemia-mimicking conditions (John et al., 1999; Contreras et al., 2002; Wang et al., 2013).

***Role of extracellular Ca^2+^***. A decrease of extracellular divalent cation concentration was one of the first conditions reported to stimulate Cx43 hemichannel opening, as introduced above. Work in a hepatoma cell line expressing Cx43 demonstrated that a decrease of the extracellular Ca^2+^ concentration triggered the uptake of Lucifer yellow, a hemichannel-permeable fluorescent dye (Li et al., 1996). Similar observations were obtained in astrocytes, where hemichannel opening was demonstrated to trigger the release of glutamate and aspartate (Ye et al., 2003). Lowering extracellular Mg^2+^ appeared to promote low Ca^2+^-triggered hemichannel opening but Ca^2+^ appeared to be the dominant factor. It is thought that normal physiological extracellular Ca^2+^ concentrations (~1.8 mM) keep non-junctional Cx hemichannels closed in the plasma membrane, until they become incorporated into gap junction channels where they open by a process called “loop gating” as a consequence of extracellular loop interactions. In general, the half-maximal effective concentration for Ca^2+^-related opening of Cx43 hemichannels is in the order of 100 μM extracellular Ca^2+^. The proposed mechanism of millimolar Ca^2+^ closure of Cx hemichannels involves an interaction of extracellular Ca^2+^ with a Ca^2+^ binding site that consists of a ring of aspartate residues at the extracellular vestibule of the hemichannel pore thereby closing the channel (Gómez-Hernández et al., 2003). Such a mechanism has been best documented for Cx32 but appears to be also plausible for the astroglial Cxs, Cx30 and Cx43. Recently, Torres et al. (2012) have elegantly exploited the extracellular Ca^2+^-sensitivity of astroglial hemichannels to trigger their opening by photo-activating a Ca^2+^ buffer (thereby increasing its Ca^2+^ affinity) added to the interstitial fluid of a hippocampal acute slice preparation (discussed further below under Section “Impact of Hemichannel-Mediated Gliotransmission on Synaptic Activity and Behavior”).

***Role of intracellular Ca^2+^***. Besides extracellular Ca^2+^, intracellular cytoplasmic Ca^2+^ also influences hemichannel function. In fact, it was found that Cx hemichannel opening triggered by a decrease of extracellular Ca^2+^ could be inhibited by ester-loading the cells with the Ca^2+^ buffer 1,2-bis(o-aminophenoxy)ethane-N,N,N^′^,N^′^-tetra-acetic acid (BAPTA), added to dampen intracellular Ca^2+^ changes (De Vuyst et al., 2006). Further investigations demonstrated that Cx32 hemichannels are activated by moderate (below 500 nM) changes in intracellular Ca^2+^ and inhibited by Ca^2+^ changes above 500 nM. Subsequent work showed that Cx43 hemichannels are also characterized by such a biphasic, bell-shaped, response to changes in intracellular Ca^2+^ concentration, in ATP release hemichannel studies as well as in dye uptake studies in glioma cells and other cell types (De Vuyst et al., 2009; De Bock et al., 2012). Recently, single channel studies of Cx43 hemichannels robustly confirmed these observations (Wang et al., 2012). Cx hemichannels are Ca^2+^ permeable (Sánchez et al., 2009, 2010; Schalper et al., 2010; Fiori et al., 2012) and the biphasic dependency on intracellular Ca^2+^ thus confers a positive feedback below 500 nM intracellular Ca^2+^ concentration (Ca^2+^-induced Ca^2+^ entry *via* Cx43 hemichannels) and negative feedback at higher concentrations. Interestingly, IP_3_ receptor channels present in the endoplasmic reticulum (ER) are Ca^2+^ release channels that have a similar bell-shaped Ca^2+^ dependency (Bezprozvanny et al., 1991). Positive and negative Ca^2+^ feedback at IP_3_ receptor channels forms the basis of Ca^2+^ oscillations, thus, it was hypothesized that Ca^2+^ feedback at Cx hemichannels could also play a role in Ca^2+^ oscillations (De Bock et al., 2012). Experimental work with various peptides targeting Cx hemichannels indeed demonstrated that inhibiting hemichannel opening, or preventing their closure in response to high intracellular Ca^2+^, blocked Ca^2+^ oscillations triggered by bradykinin. Thus, Cx hemichannels constitute a putative gliotransmitter release pathway. Also, they may contribute to Ca^2+^ oscillations in astrocytes and thereby potentially lead to gliotransmitter release *via* other Ca^2+^-dependent pathways (Oliet and Mothet, 2006; Zorec et al., 2012). In brain endothelial cells, it was found that Cx hemichannel blocking peptides prevented the opening of the blood–brain barrier, triggered by bradykinin, by inhibiting Ca^2+^ oscillations in the endothelial cells (De Bock et al., 2011). Below, we discuss in more detail some peptide-based hemichannel inhibitors.

#### Influence of monovalent cations

Elevations in extracellular K^+^ shift the activation potential of Panx1 hemichannels to a more physiological range. Panx1 hemichannels expressed in astrocytes might serve as K^+^ sensors for changes in the extracellular milieu such as those occurring under pathological conditions (Suadicani et al., 2012). This type of regulation might also occur under physiological conditions with high neuronal activity. Thus, elevations in extracellular K^+^ of the magnitude occurring during periods of high neuronal activity have been proposed to affect the intercellular signaling among astrocytes (Scemes and Spray, 2012). Accordingly, the gliotransmitter release in the hippocampus in response to high neuronal activity is sensitive to P2 receptor and Panx1 channel blockers (Heinrich et al., 2012). Cx30 gap junction channels have recently been demonstrated to be regulated by the concentration of external K^+^ (Roux et al., 2011); whether astroglial Cx hemichannels are influenced by the extracellular K^+^ concentration is currently not known.

#### Influence of pH

Under pathological conditions, the intracellular and extracellular pH may display abrupt changes. Related to this issue and in the presence of extracellular divalent cations, Cx43 hemichannels are opened by alkaline pH applied at the extracellular side and preferentially closed at physiologic pH (Schalper et al., 2010). The activity of Cx45 hemichannels recorded at positive membrane potentials and in the absence of extracellular Ca^2+^ is drastically reduced by acidification (Valiunas, 2002). In addition, the pH sensitivity might be potentiated by protonated aminosulfonates (Bevans and Harris, 1999), such as taurine that could be in millimolar concentration in the cytoplasm of several cell types including astrocytes (Olson, 1999). This effect is Cx specific; Cx26 but not Cx32 hemichannels are closed by aminosulfonates at constant pH (Bevans and Harris, 1999). At the ultrastructural level (high-resolution atomic force microscopy), Cx26 hemichannels are closed at pH < 6.5 [4-(2-hydroxyethyl)-1-piperazineethanesulfonic acid (HEPES) buffer] and opened reversibly by increasing the pH to 7.6 (Yu et al., 2007). Moreover, molecular studies have revealed that Cx26 hemichannel closure induced by taurine requires the interaction between the cytoplasmic loop and the C-terminal (CT; Locke et al., 2011).

#### Influence of mechanical forces

Mechanical stress is another condition that might be relevant under pathological situations. For example, after brain trauma the extracellular medium becomes hypertonic due to the release of intracellular solutes to the extracellular microenvironment (Somjen, 2002). In astrocytes, hypertonicity induces glutamate release through Cx43 hemichannels (Jiang et al., 2011). This response could be mediated by integrin α5β1 (Batra et al., 2012), a RhoA GTPase and the contractile system (Ponsaerts et al., 2010). This sensitivity is likely to result from the interaction between negatively charged amino acid residues of the CT end (Asp278 and Asp279) and a domain of the intracellular loop (D’Hondt et al., 2013). Activation of Panx1 channels by mechanical stress is not present in all cell types (Bao et al., 2004a; Reyes et al., 2009), suggesting that it should be tested in glial cells in order to validate its possible relevance in these cells.

#### Influence of extracellular factors and signals

The functional state of glial Cx hemichannels and Panx1 channels is actively regulated by extracellular signals. In primary cultures of mouse astrocytes, stomatin inhibits Panx1-mediated whole cell currents by interacting with its CT (Zhan et al., 2012). In contrast, pro-inflammatory agents such as the β-amyloid peptide increase the surface expression and activity of Cx43 hemichannels and Panx1 channels in microglia and Cx43 hemichannels in astrocytes (Orellana et al., 2011c). Moreover, in brain astrocytes, epidermal growth factor (EGF) and FGF-2 inhibit Cx hemichannel activity *via* the mitogen-activated protein kinase cascade, and the effect of the growth factors is reversed by interleukin-1β (IL-1β; Morita et al., 2007). In contrast, pro-inflammatory cytokines [tumor necrosis factor alpha (TNF-α) and IL-1β] released by LPS-activated microglia increase the activity of astroglial Cx43 hemichannels via p38 kinase (Retamal et al., 2007b; Orellana et al., 2011c) and this response is inhibited by activation of CB1 receptors by synthetic cannabinoid agonists (Froger et al., 2009). The orchestrated involvement of Cx hemichannels and Panx1 channels has also been found in other experimental preparations. In spinal cord astrocytes, FGF-1 leads to vesicular ATP release followed by sequential activation of Panx1 and Cx43 hemichannels (Garré et al., 2010). Moreover, up-regulation of astroglial Panx1 channels and Cx43 hemichannels has been found in brain abscess (Karpuk et al., 2011).

Cx43 hemichannels expressed by tanycytes, glial cells present in the hypothalamus, open in seconds after exposure to physiological concentrations of extracellular glucose. This response is mediated by the sequential activation of glucosensing proteins (Orellana et al., 2012a), a response that is absent in cortical astrocytes (Orellana et al., 2010). However, high glucose levels enhance the increase in astroglial Cx43 hemichannel activity induced by hypoxia-reoxygenation (Orellana et al., 2010).

As a result of cell interaction with extracellular signals or conditions that affect the energy supply, intracellular metabolic pathways involving levels of free Ca^2+^ concentration, activity of protein kinases and phosphatases, as well as levels or activity of intracellular redox molecules, are affected. Therefore, drastic reduction in ATP levels are expected to favor the dephosphorylated state of Cx43 hemichannels and thus, they could present an increased activity (Bao et al., 2007). Moreover, the lack of energy leads to a rise in intracellular free Ca^2+^ that favors the generation of reactive oxygen and nitrogen species including NO. The latter induce rapid nitrosylation of Cx43 and opening of hemichannels (Retamal et al., 2007b; Figueroa et al., 2013). This mechanism might partially explain the increase in Cx43 hemichannel activity observed in astrocytes under ischemia-like conditions since free radical scavengers, dithiothreitol, a sulfhydryl (SH) reducing reagent, and high intracellular levels of reduced glutathione revert the hemichannel activity to levels found in normal cells (Contreras et al., 2002; Retamal et al., 2007b; Orellana et al., 2010). Finally, extracellular ATP mediates the ischemic damage to oligodendrocytes and is partially explained by the activation of Panx1 channels (Domercq et al., 2010). In contrast to Cx43 hemichannels, *S*-nitrosylation of Panx1 channels reduces their activity (Lohman et al., 2012), suggesting that their opening might be relevant only under conditions where NO is overcome by -SH reducing agents such as reduced glutathione.

## HEMICHANNEL BLOCKERS – MIMETIC PEPTIDES AND OTHERS

Specifically targeting unapposed hemichannels is difficult because they are composed of the same Cx building blocks as gap junction channels. As a consequence, most gap junction blockers also inhibit hemichannels (see Giaume and Theis, 2010). Moreover, many Cx hemichannel/gap junction channel blockers also block Panx channels. Gap junction blockers include glycyrrhetinic acid and its derivative carbenoxolone, long-chain alcohols like heptanol or octanol, halogenated general volatile anesthetics like halothane, fatty acids like arachidonic acid and oleic acid, fatty acid amides like oleamide and anandamide, and fenamates like flufenamic acid, niflumic acid, or meclofenamic acid (reviewed in Bodendiek and Raman, 2010). Most of these substances have been used in the past to block hemichannels but the results obtained can only be unequivocally interpreted in terms of hemichannels when the contribution of gap junction channels is negligible or absent. Substances like the trivalent ions gadolinium (Gd^3+^) and lanthanum (La^3+^) block hemichannels without inhibiting gap junction channels or Panx1 channels, but these ions also inhibit other channels including maxi-anion channels (Liu et al., 2006) and Ca^2+^ channels (Mlinar and Enyeart, 1993). A possible way to differentiate between Cx hemichannels and Panx channels is the use of long-chain alcohols that block Cx hemichannels but have only a very small effect of Panx channels. In contrast, low concentration (5–10 μM) of carbenoxolone blocks Panx1 channels and only has a minor inhibitory effect on Cx hemichannels (Schalper et al., 2008a). However, all these compounds are not specific and in addition to their inhibitory effects on Cx and Panx channels (including gap junctions channels and hemichannels) they also affect other neuronal and glial membrane properties which limits their use (see Giaume and Theis, 2010).

**FIGURE 2:**
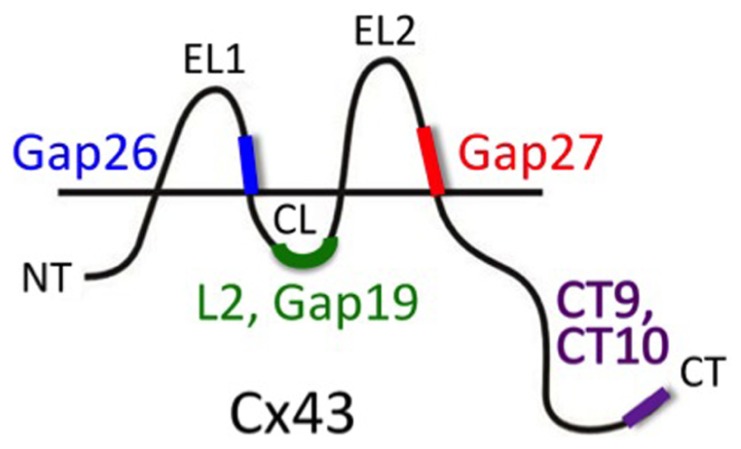
**Peptide tools to interfere with Cx hemichannel function**. Gap26 and Gap27 are composed of sequences located on the extracellular loops 1 (EL1) and 2 (EL2), respectively (amino acids 64–76 and 201–210, respectively) of Cx43 (mouse). These EL-mimetic peptides first inhibit hemichannels and with some delay also gap junction channels. The sequences they mimic are well conserved between different Cxs and peptides based on Cx43 sequences could also inhibit channels composed of other Cxs (e.g., Cx37). L2 peptide is identical to the L2 domain on the cytoplasmic loop (CL; amino acids 119–144). Gap19 is a nonapeptide located within the L2 domain (amino acids 128–136). L2 and Gap19 peptides block Cx43 hemichannels without blocking gap junctions and without blocking Cx40 hemichannels or Panx1 channels (other Cxs still need to be tested). CT10 and CT9 are the last 10 and last 9 amino acids of the C-terminal end (CT; amino acids 373–382 and 374–382, respectively). CT9/CT10 peptides remove the closure of Cx43 hemichannels with high micromolar cytoplasmic Ca^2+^ concentration and thus stimulate the opening of hemichannels. This particular effect of the CT-peptides is independent of the last amino acid (isoleucine 382) that is involved in linking Cx43 to scaffolding proteins involving ZO-1 interactions (the amino acid sequence 374–381 has the same effect as CT9/CT10; De Bock et al., 2012).

A more specific targeting of Cx channels is to be expected, at least in principle, from mimetic peptides of Cx proteins. Peptides identical to certain sequences on the Cx protein have been extensively used to interfere with the Cx channel function (**Figure 2**). The first Cx mimetic peptides that were introduced in the 1990s were identical to specific domains on the extracellular loops (first or second extracellular loop) of the Cx protein. The sequences mimicked were located in domains thought to be involved in the docking of two hemichannels during formation of a full gap junction channel (Warner et al., 1995). Exogenous addition of peptides mimicking parts of these domains were hypothesized to interact with yet unknown extracellular loop domains, thereby preventing extracellular loop interactions of apposed hemichannels during docking and thus hindering gap junction channel formation. These domains, known as Gap26 and Gap27, are present on the first and second extracellular loops, respectively. Accordingly, Gap26 and Gap27 peptides were indeed found to inhibit gap junctional coupling (Evans and Boitano, 2001). Interestingly, subsequent work demonstrated that Gap26 and Gap27 peptides also inhibited unapposed hemichannels with which these peptides are supposed to interact (Braet et al., 2003; Evans et al., 2006, 2012; Desplantez et al., 2012; Wang et al., 2012). Currently, clear evidence that these peptides indeed interact with the extracellular loops is only available for Gap26 (Liu et al., 2006). Nevertheless, both Gap26 and Gap27 rapidly inhibit hemichannels within minutes, followed by a somewhat delayed inhibition of gap junction channels, often in the range of hours, with some exceptions (Matchkov et al., 2006). The exact mechanism of hemichannel block is currently not known. It is known that Gap26/27 influence voltage-dependent gating (Wang et al., 2012) but this does not explain their inhibitory action on hemichannels opened by triggers other than voltage. It has been suggested that these peptides inhibit hemichannels by just blocking the pore because of steric hindrance effects (Wang et al., 2007). However, this has been carefully checked and it was found that this only occurs at very high (1 mM and above) concentrations (Wang et al., 2012). Although Gap26/27 peptides proved to be interesting tools to inhibit Cx hemichannels, their delayed inhibition of gap junction channels remains a serious drawback. Moreover, Gap26/27 peptides also show rather limited specificity toward different Cx types. Indeed, the extracellular loop domains mimic sequences that show pronounced homology between different Cx proteins. Thus, Gap27 directed against Cx43 in astrocytes is also known to inhibit channels or hemichannels composed of Cx37, a vascular Cx present in brain blood vessels (Martin et al., 2005).

Besides peptides, antibodies have also been used to target the free extracellular loops of unapposed Cx hemichannels resulting in their inhibition. These tools offer additional advantage because they allow both functional blocking of the hemichannels and visualizing their distribution (Hofer and Dermietzel, 1998; Clair et al., 2008; Riquelme et al., 2013). However, currently single channel electrophysiological data showing hemichannel block by an antibody directed against an extracellular epitope is only demonstrated for Cx43 hemichannel activity induced in β-amyloid-treated astrocytes (Orellana et al., 2011c). Additionally, the specificity toward effects on other Cxs or Panxs still needs to be determined.

To improve specificity toward different Cxs, peptides have been devised that should target sequences on the intracellular domains of the Cx protein. The intracellular sequences are poorly conserved and very different between different Cxs. The L2 peptide is identical to a sequence on the cytoplasmic loop of Cx43. This peptide is known to prevent the closure of gap junction channels by binding to the CT tail, thereby preventing interaction of the CT tail with the cytoplasmic loop of Cx43 where the L2 domain is located (Seki et al., 2004; Hirst-Jensen et al., 2007 and references therein). Conceptually, interaction between the CT tail and the L2 domain is thought to block the gap junction channel pore upon exposure to acidification according to a particle-receptor model, and exogenous addition of L2 peptide prevents this interaction and its associated channel closure. Unexpectedly, it was found that the L2 peptide inhibited Cx43 hemichannels, based on ATP release studies, indicating that this peptide has distinct effects on unapposed hemichannels and those incorporated into gap junctions (Ponsaerts et al., 2010). Further work with Gap19, a mimetic nonapeptide located within the Cx43 L2 domain, demonstrated a similar action; it inhibited single channel hemichannel currents and ATP release via Cx43 hemichannels while it did not influence gap junctional coupling after 30 min and stimulated coupling after 24 h (Wang et al., 2013). Moreover, the effect appeared to be specific for Cx43 as Gap19 had no effect on Cx40 hemichannels or Panx1 channels. While the effects of L2 and Gap19 are understood at a molecular operational scale, the full details of why preventing interactions between the CT tail and the cytoplasmic loop have such different effects on hemichannels as compared to gap junction channels still needs further clarification. However, distinct effects on hemichannels and gap junction channels have been reported for other Cx channel influencing molecules or conditions; for example, basic FGF (bFGF) and cytokines like TNF-α or IL-1β have all been demonstrated to promote Cx43 hemichannel function while inhibiting gap junctions (De Vuyst et al., 2007; Retamal et al., 2007a). Given the specific actions of L2 and Gap19 peptides at the level of both Cx types and channel types (hemichannels *versus* gap junction channels), these peptides offer promising possibilities to explore the role of astroglial Cx43 hemichannels in the complex environment of intact neural tissues, *ex vivo* or *in vivo*. Recent work has, for example, demonstrated that injection of L2 peptide in the rat amygdala strongly suppresses fear memory (Stehberg et al., 2012; discussed further below under Section “Impact of Hemichannel-Mediated Gliotransmission on Synaptic Activity and Behavior”). Equally interesting with respect to identifying the role and functions of Cx43 hemichannels is the G138R mutation of Cx43 in which a Gly at position 138 is replaced by an Arg. This is one of the several mutants found in the inherited human disease oculodentodigital dysplasia (ODDD). Importantly, G138R mutant Cx43 is characterized by a loss-of-function of gap junctional coupling and a gain-of-function of hemichannels (Dobrowolski et al., 2007, 2008). Thus, this particular mutant Cx43 can be used to stimulate Cx43 hemichannel function as a complementary experimental approach to hemichannel inhibition. Recently, Torres et al. (2012) have exploited such an approach to investigate the role of hemichannels in glial–neuronal communication.

## ROLES OF GLIAL HEMICHANNELS IN HEALTH AND DISEASE

### ASTROGLIAL Ca^2+^ WAVES

Initially, gap junction channels were demonstrated to constitute the pathway allowing the propagation of intercellular Ca^2+^ signaling between astrocytes, termed as Ca^2+^ waves (Finkbeiner, 1992; Venance et al., 1995). Then, after the discovery of an external component that also contributes to this interglial signaling process (Hassinger et al., 1996), the involvement of Cx43 hemichannels and ATP release was identified (Cotrina et al., 1998; Stout et al., 2002). There is a consensus on the interpretation of these two sets of *in vitro* studies and it is admitted that both Cx channel functions are contributing. Indeed, they likely participate in various proportions to Ca^2+^ waves depending on the brain structure, the mode of stimulation to trigger the waves, the age and the *in vitro* conditions (Scemes and Giaume, 2006; Leybaert and Sanderson, 2012). In addition, several of these points could also account for the alternative proposal claiming that in astrocytes the external component is not supported by Cx43 hemichannels but by Panx1 channels (Suadicani et al., 2006; Scemes et al., 2007). This point of controversy remains to be clarified in the future although it is likely that both might participate in various proportions under different conditions.

More recently, several reports have confirmed that Ca^2+^ waves in astrocytes are present *in vivo* in the normal brain (Hoogland et al., 2009; Kuga et al., 2011; Mathiesen et al., 2013) as well as in pathological situations (Kuchibhotla et al., 2009; Mathiesen et al., 2013). As expected, the occurrence, amplitude and extent of *in vivo* Ca^2+^ waves are much more limited compared to the initial *in vitro* observations. However, as Ca^2+^ signaling is considered as the mode of cellular excitability in astrocytes, such waves certainly play a role in the spatial and temporal features of neuroglial interactions. As a consequence, this means that in many cases dynamic neuroglial interactions are not limited to the sole tri-partite synapse but also occur at a more integrated level involving larger cerebral areas (Giaume et al., 2010). This situation is also likely true at the gliovascular interface where there is a high level of Cx expression between astroglial perivascular endfeet (Yamamoto et al., 1990; Simard et al., 2003; Rouach et al., 2008) and where Ca^2+^ waves propagate from one initial endfoot to its neighbors (Mulligan and MacVicar, 2004). Such features might optimize the contribution of astrocytes to the control of the cerebral blood flow. However, while a role of Cx43 gap junction channels/hemichannels in pial arteriole dilation has been demonstrated *in vivo* for the glia limitans in response to sciatic stimulation (Xu and Pelligrino, 2007), a similar mechanism remains to be investigated for perivascular astrocytes within the brain parenchyma.

### HEMICHANNELS AS Ca^2+^ CHANNELS

As indicated above, glial cells express several Cxs and out of these at least four of them (Cx26, Cx30, Cx32, and Cx43) have been demonstrated to form functional hemichannels (Li et al., 1996; Rhee et al., 1996; Valiunas and Weingart, 2000; Ripps et al., 2004), and at least Cx26, Cx32, and Cx43 serve as a membrane pathway for cell (Sánchez et al., 2009, 2010; Schalper et al., 2010) or liposome (Schalper et al., 2010; Fiori et al., 2012) Ca^2+^ inflow. Of course, brief opening of Cx hemichannels could serve to transiently increase the intracellular Ca^2+^ concentration that could affect a broad variety of physiological cell functions. For example, Cx43 hemichannels contribute to maintain higher intracellular levels of free Ca^2+^ in cells treated with FGF-1 that proliferate and remain healthy (Schalper et al., 2008b). However, sustained hemichannel opening could promote deleterious results, including cell death (Decrock et al., 2009, 2011), but the bell-shaped action of intracellular Ca^2+^ might prevent this ending process. Panx1 has also been detected in glial cells, as mentioned above (Iglesias et al., 2009; Domercq et al., 2010; Orellana et al., 2012b). This glycoprotein has been shown to form channels in the ER permeable to Ca^2+^ (Vanden Abeele et al., 2006; D’Hondt et al., 2011) but the permeability to Ca^2+^ of Panx1 channels present in the cell surface remains elusive. Moreover, direct demonstration of either Cx- or Panx-based channels as a Ca^2+^ membrane pathway in any glial cell type remains to be demonstrated.

### ALTERNATIVE PATHWAY FOR GLUCOSE ENTRY IN INFLAMMATORY ASTROCYTES

Through the so-called astrocyte–neuron lactate shuttle, astrocytes participate in an activity-dependent manner to the uptake of glucose from the blood supply and the delivery of lactate to neurons (Pellerin and Magistretti, 2012). In normal conditions, glucose and lactate transporters are the membrane elements that support this astroglial contribution to brain metabolism. While glucose is well known to be essential for correct brain function, its homeostasis is altered by inflammatory conditions (Allaman et al., 2011). *In vitro*, an inflammatory situation for astrocytes can be mimicked either by co-culturing them with microglia selectively activated by the endotoxin LPS or by directly treating them with pro-inflammatory cytokines, such as TNF-α and IL-1β. Interestingly, in both cases these treatments induce an opposite regulation of Cx43 channels at least in cultured astrocytes: a decrease in gap junctional communication and an activation of hemichannels (Retamal et al., 2007a). In these conditions Cx43 hemichannels were shown to allow the influx of 2-(*N*-(7-nitrobenz-2-oxa-1,3-diazol-4-yl)amino)-2-deoxyglucose (2-NBDG), a glucose fluorescent analog, demonstrating that reactive or inflamed astrocytes can take up more glucose via Cx43 hemichannels when their metabolic coupling through gap junction channels is reduced (Rouach et al., 2008). Such opening of Cx43 hemichannels may represent an alternative metabolic pathway for glucose entry in astrocytes during pathological conditions associated with an inflammation status (Sofroniew and Vinters, 2010). Interestingly, this observation identifies a pathway that could contribute to the increase of glucose uptake found in several uncoupling situations (Tabernero et al., 2006); this was correlated with the up-regulation of GLUT-1 (glucose transporter 1) expression and to the induction of the expression of GLUT-3, an isoform that is not normally expressed in astrocytes, as well for type I and type II hexokinases, respectively (Sánchez-Alvarez et al., 2004). However, the respective contribution of hemichannels and glucose transporters in inflammatory conditions remains to be established. Because the permeability of the blood–brain barrier is increased during the inflammatory response (Schnell et al., 1999), more glucose coming from the circulation could become available to the brain. Thus, in addition to transporters, the entry of glucose through Cx43 hemichannels could contribute to an increase of lactate formation by astrocytes that are adapted for anaerobic metabolism. Finally, the opposite regulation of Cx43 channels (Retamal et al., 2007a) may lead to a failure in intercellular glucose trafficking (Rouach et al., 2008) that could be compensated for by the increase in glucose influx through Cx43 hemichannels in individual astrocytes. Moreover, although not demonstrated yet, Cx43 hemichannels could also support lactate efflux to feed neurons since Cx43 gap junction channels in astrocytes are permeable to lactate (Tabernero et al., 1996; Rouach et al., 2008). As a whole, Cx43 hemichannel activation should certainly contribute to modify the metabolic status of reactive astrocytes, a situation that should be taken into account when considering the role of astroglia in brain inflammation.

### IMPACT OF HEMICHANNEL-MEDIATED GLIOTRANSMISSION ON SYNAPTIC ACTIVITY AND BEHAVIOR

By analogy with the concept of a neurotransmitter, the term “gliotransmitter” has been proposed to name active molecules that are released by astrocytes and regulate neuronal activity as well as synaptic strength and plasticity (Haydon and Carmignoto, 2006). There are mainly three candidates that are proposed to play this role: glutamate, ATP, and D-serine. All of them can be released by a Ca^2+^-dependent vesicular release mechanism in astrocytes (Zorec et al., 2012). However, at least glutamate and ATP have been demonstrated to permeate Cx43 hemichannels in astrocytes (Ye et al., 2003; Kang et al., 2008; Orellana et al., 2011b; Iglesias and Spray, 2012) as well as Panx1 channels in microglia (Orellana et al., 2011c), thus Cx43 and Panx1 channels could also play a role in gliotransmission. Recently, the activation of Cx43 hemichannels in astrocytes was shown to impact synaptic activity and plasticity (Stehberg et al., 2012; Torres et al., 2012). The first evidence comes from hippocampal acute slices in which a decrease in extracellular Ca^2+^ concentration is generated by the local photolysis of a photolabile Ca^2+^ buffer. This opens Cx43 hemichannels in astrocytes through which ATP is released and activates P2Y_1_ purinergic receptors on a subset of inhibitory interneurons, initiating the generation of spikes by interneurons that in turn enhances synaptic inhibition at glutamatergic synapses (Torres et al., 2012). A similar neuroglial loop of interaction acting as a negative feedback mechanism is expected to occur during excessive glutamatergic activity that is associated with a decrease in extracellular Ca^2+^ concentration (Rusakov and Fine, 2003). The second set of evidence comes from *in vivo* experiments in which Cx43 hemichannels were inhibited (Stehberg et al., 2012). In this study, the rat basolateral amygdala was microinfused with the TAT-L2 peptide that selectively inhibits Cx43 hemichannels assumed to be only present in astrocytes. Such *in vivo* blockade of Cx43 hemichannels during memory consolidation induces amnesia for auditory fear conditioning, as assessed 24 h after training, without affecting short-term memory, locomotion, or shock reactivity. Moreover, the amnesic effect is transitory, specific for memory consolidation. Learning capacity recovers after co-infusion of the Cx43 hemichannel blocker and a mixture of gliotransmitters including glutamate and ATP. These observations suggest that gliotransmission mediated by Cx43 hemichannels is necessary for fear memory consolidation at the basolateral amygdala. Thus, the study of Stehberg et al. (2012) represents the first *in vivo* demonstration of a physiological role for astroglial Cx43 hemichannels in brain cognitive function. Finally, an *in vivo* contribution of Panx1 channels to cognition has recently been addressed by using a Panx1 knock out mouse (Prochnow et al., 2012). This constitutive knock out mouse was found to have increased excitability and potently enhanced early and persistent long-term potentiation (LTP) responses in the CA1 hippocampal region with additionally impaired spatial memory and object recognition memory. However, while the expression of Panx1 is well documented in hippocampal neurons (MacVicar and Thompson, 2010), its detection at the membrane of astrocytes is still debated (Iglesias et al., 2009; but see Orellana et al., 2010, 2011c). Consequently, it is premature to state that these Panx1-dependent changes in hippocampal models of learning and memory have an astroglial origin.

### NEURONAL DEATH AND BRAIN PATHOLOGIES

While there are now a number of reports indicating that the expression of glial Cxs changes in many brain pathologies and injuries (Giaume et al., 2010), this remains to be investigated in detail for Panxs. On this basis, the question of Cx channels contributing to neuronal death and brain diseases has been debated for many years. Indeed, opposite perspectives regarding the roles of glial Cxs in neuronal death have been discussed and reviewed (see Kirchhoff et al., 2001; Rouach et al., 2002; Perez Velazquez et al., 2003; Nakase and Naus, 2004; Farahani et al., 2005; Giaume et al., 2007; Orellana et al., 2009). In general, this duality was attributed to differences in (i) the experimental models (*in vitro*, *ex vivo*, and *in vivo*) or the protocols used to induce neuronal death, (ii) the mode (pharmacology *versus* transgenic animals) of Cx channel inhibition selected, (iii) the type of neuronal death or pathology investigated, and (iv) the time points considered. However, retrospectively the opposite roles attributed to Cx channels could be simply due to the fact that for a long time this question was solely taking into account the gap junctional function of Cxs. Indeed, in most cases, interpretation of the data was not considering the hemichannel and channel functions of Cxs and Panxs, respectively. Nevertheless, the development of compounds and transgenic animals that discriminate between Cx gap junction channels, Cx hemichannels and Panx channels (see above) has allowed identification of their respective contribution in various *in vitro* and *ex vivo* models of brain pathologies (see Kielian, 2008; Orellana et al., 2009; Bennett et al., 2012). Although hemichannels have been reported to be involved in several brain pathologies or injuries (reviewed in Bennett et al., 2012) as well as infection (Karpuk et al., 2011; Xiong et al., 2012), we will illustrate this situation by two recent examples that demonstrate the impact of glial Cx hemichannel and Panx channel activation in cells on neuronal death.

Modifications in the pattern of Cx and Panx expression in glia are associated with phenotypic changes observed during neuroinflammation and the “reactive gliosis” that is a common feature of most brain diseases (see Sofroniew and Vinters, 2010). Indeed, activated microglia and reactive astrocytes exhibit changes in their respective pattern of expression for Panx1 and Cx43 as well as in their hemichannel function leading to neuronal damage and death (Bennett et al., 2012; Orellana et al., 2012b). When an inflammatory context is mimicked *in vitro* by using LPS treatment, Cx43 hemichannel activity is triggered in astrocytes through the production of TNF-α and IL-1β by activated microglia. These events are not observed when astrocytes are cultured from Cx43 knock out mice (Retamal et al., 2007a). Furthermore, in neuron–astrocyte co-cultures the *N*-methyl-D-aspartate (NMDA)-induced neuronal excitotoxicity is reinforced by TNF-α + IL-1β treatment, while such a potentiating effect is not observed in cultures of neurons alone treated with the two cytokines (Froger et al., 2010). This observation suggests that the well-known protective role of astrocytes against excitotoxicity (Chen and Swanson, 2003) is impaired under pro-inflammatory conditions when astroglial Cx43 hemichannels are activated. Neuron–astrocyte co-cultures, made with astrocytes from Cx43 knock out and neurons from wild-type mice, revealed that the NMDA excitotoxicity is not reinforced by the pro-inflammatory treatment applied on these co-cultures made with Cx43-deficient astrocytes (Froger et al., 2010). This result suggests that astroglial Cx43 is involved in the potentiated neurotoxicity induced by pro-inflammatory treatments. Interestingly, application (<30 min) of mimetic peptides (Gap26, Gap27) that inhibit Cx43 hemichannels and have no effect on Cx43 gap junctional communication (Retamal et al., 2007a), prevents the potentiated neurotoxicity response induced by pro-inflammatory cytokines (Froger et al., 2010; Orellana et al., 2012b).

Connexin hemichannels might also play a role in the pathological context of neurodegenerative diseases. Indeed, it is becoming increasingly clear that glial cells are critical players in the progressive neurodegeneration of Alzheimer’s disease (Kraft et al., 2013). Among others, the amyloid cascade represents one of the key pathways involved in the pathogenesis of the disease (see Pimplikar, 2009). Using separate primary cultures and co-cultures of microglia, astrocytes, and neurons it was shown that treatment with the β-amyloid peptide (Aβ) first activates microglia, resulting in the opening of Cx43 hemichannels and Panx1 channels in these cells. Such channel activations allow the release of glutamate and ATP that impair neuronal survival. Aβ-activated microglia also produces TNF-α and IL-1β that, as indicated above, induce Cx43 astroglial hemichannel activation that again results in the release of glutamate and ATP (Orellana et al., 2011c). *In fine* these gliotransmitters originating from glial cells trigger neuronal damage and induce their death. Thus, such a cascade of Cx glial hemichannel and Panx channel activation could partially account for the progression of the neurodegenerative disease. These *in vitro* and *ex vivo* observations could be related to the changes in Cx43 expression observed in reactive astrocytes that contact amyloid deposit in a murine model of Alzheimer’s disease (Mei et al., 2010) and in brain from Alzheimer’s patients (Nagy et al., 1996; Koulakoff et al., 2012).

## CONCLUDING REMARKS

During the last decade our view and understanding of the structure (Maeda and Tsukihara, 2011), mutations (Pfenniger et al., 2011), properties (Rackauskas et al., 2010), and biological roles (Kar et al., 2012) of Cx channels has been incredibly enlarged, and to some extent this is also true for Panx channels (MacVicar and Thompson, 2010; Penuela et al., 2013). Interestingly, in glial cells this feature is exemplary because in addition to their well-documented molecular support for their two channel functions, non-channel functions have been also identified for Cxs, including cell adhesion mechanisms involved in radial migration of cortical neurons (Elias et al., 2007; Cina et al., 2009), modulation of P2Y purinergic receptors signaling (Scemes, 2008), regulation of cytoskeletal dynamics (Olk et al., 2010) and control of gene expression (Iacobas et al., 2004). Such a view could even be further enlarged by the recent report of another new class of membrane protein called calcium homeostasis modulator CALHM1 that shares structural similarities with Cx, Panx, and innexin channels and functional properties similar to hemichannels, such as ATP release in taste transduction (Siebert et al., 2013; Taruno et al., 2013).

In the present review, we have focused on a Cx/Panx-based channel function which 10 years ago was only sparsely considered in the central nervous system and in neuroglial interactions. Since then, the identification of external and intracellular signals that trigger the activation of hemichannels, the understanding of their biophysics and their regulation have provided a better understanding of the roles that glial Cxs and Panxs play in healthy and diseased brains. Indeed, as reported above, there is now strong evidence to state that Cx and Panx channels in glia have an impact on neuronal activity and survival. However, there are at least two aims that remain to be achieved in order to determine whether and how the contribution of glial Cxs and/or Panxs is causal or secondary in normal and pathological situations. One is to distinguish what is due to gap junction channels *versus* hemichannels, the other is to discriminate between Cx hemichannels and Panx channels. To achieve these goals it is essential to have available pharmacological and genetic tools with glial cell type, Cx type, and Panx type specificities (see Giaume and Theis, 2010). Through a simple pharmacological approach this is unlikely to be achieved since Cx and Panx channels are sensitive to the same agents so far described. However, strategies using short-term treatment with mimetic peptides (Evans et al., 2012) or antibodies targeting extracellular domains (Riquelme et al., 2013) could be developed for all the glial Cxs/Panxs and their specificity demonstrated. In contrast, due to the cytoplasmic location of their two channel ends, gap junction channels are likely less easy to target by a pharmacological strategy. Another more accurate approach consists in the development of transgenic mice with appropriate mutations that prevent channel pairing without affecting hemichannel activity or that block one but not the other channel function. Alternatively, small interfering RNA (siRNA) and transgenic/viral techniques have been used with some success (see Giaume and Theis, 2010). But here again, the expression of multiple Cxs in glial cells makes the task difficult with genetic and molecular approaches. A recent report by Nedergaard’s group of an enhanced synaptic plasticity (LTP) due to the engraftment of human glial progenitors into immunodeficient mice (Han et al., 2013) gives weight to achieve such difficult challenges. Indeed, upon maturation in these grafted mice, human astrocytes were shown to be coupled to host astrocytes, suggesting a contribution of Cx channels in neuroglial interactions during synaptic plasticity. In line with such contribution, double Cx43/Cx30 knock out is associated with reduced LTP (Pannasch et al., 2011). Also, the observation of an up-regulation of astroglial Cxs in reactive astrocytes contacting amyloid plaques in a transgenic murine model of Alzheimer’s disease has been validated in human patient brain samples (Nagy et al., 1996; Koulakoff et al., 2012), giving another example of the value in searching for strategies that block Cx channels.

## Conflict of Interest Statement

The authors declare that the research was conducted in the absence of any commercial or financial relationships that could be construed as a potential conflict of interest.
